# Proof-of-Concept Development of a Bioelectric Biosensor Using Arduino for Monitoring Dopaminergic Response in Neuroblastoma Cells

**DOI:** 10.3390/mi16080951

**Published:** 2025-08-19

**Authors:** Magdalene Pappa, Spyridon Kintzios

**Affiliations:** Laboratory of Cell Technology, Department of Biotechnology, Agricultural University of Athens, Iera Odos 75, 11855 Athens, Greece; stud312031@aua.gr

**Keywords:** Arduino, bioelectric biosensor, bioelectric recognition assay (BERA), cellular resistance, dopamine, gel immobilization, neuroblastoma cells

## Abstract

This study presents the proof-of-concept design and preliminary implementation of a bioelectric biosensor based on an Arduino platform for real-time monitoring of gel-immobilized N2a neuroblastoma cells using dopamine as a model neurotransmitter. The sensor operates on the principle of bioelectric recognition assay (BERA), and uses a two-electrode set-up as a simple, cost-efficient way to capture electrophysiological responses following dopamine exposure, while at the same time mimicking the in vivo cellular environment. Cellular ohmic resistance was assessed under increasing dopamine concentrations and temperatures (24 °C and 37 °C). The results showed that temperature significantly affected cell responses to increasing dopamine concentrations, possibly because of differences in dopamine diffusion in gel, which may in turn have affected membrane polarization and overall cell electric resistance. Pending further testing against a wider range of dopamine concentrations along with various dopamine agonists/antagonists, as well as optimization in terms of specificity, selectivity, and sensitivity, the biosensor could be applied in bioscreening and neuropharmacological studies in a user-friendly, scalable way.

## 1. Introduction

**Figure 1:**
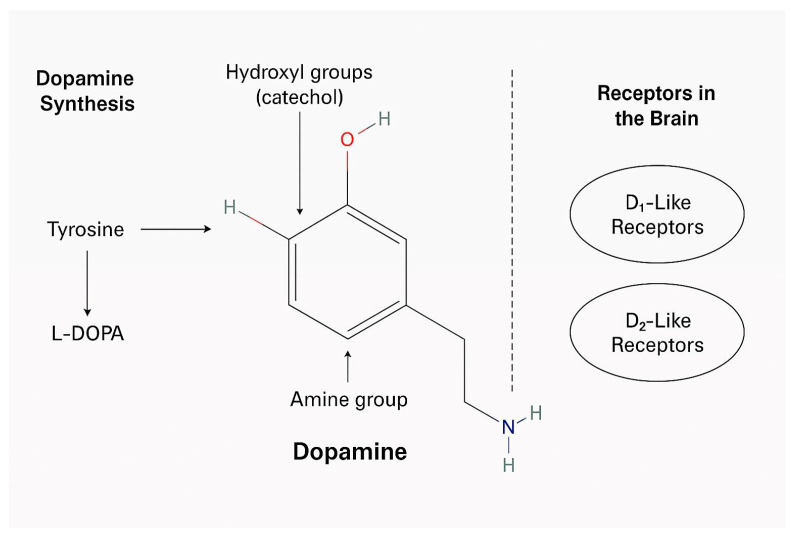
The molecular structure and synthesis of dopamine, and key dopamine receptors in the brain.

Dopamine is a crucial catecholamine neurotransmitter (Figure 1) that plays multifaceted roles in the central nervous system, modulating motor control, motivation, cognition, and reward-based behavior [1]. Its function in the mesolimbic and mesocortical pathways underpins emotional processing and executive functions, with alterations in dopaminergic signaling contributing to the pathophysiology of several psychiatric disorders, including schizophrenia, bipolar disorder, depression, and attention-deficit hyperactivity disorder (ADHD) [2,3].

Due to the critical role of dopamine in mental health, the precise quantification of dopamine levels in biological samples is essential for neurochemical research and clinical diagnostics. Conventional and biosensor-based analytical techniques for dopamine detection include high-performance liquid chromatography (HPLC) coupled with electrochemical or fluorescence detection [4], as well as capillary electrophoresis [5], enzyme-linked immunosorbent assays (ELISAs) [6], and electrochemical techniques [7]. However, all these methods lack portability and are unsuitable for rapid, point-of-care mental health diagnostics, thus motivating the development of novel biosensor-based technologies capable of real-time, cost-effective, and selective dopamine detection to support psychiatric research and personalized treatment strategies [8].

Beyond detection, real-time monitoring of dopamine–neural-cell interactions is critical for understanding synaptic transmission dynamics, receptor pharmacology, and neurotoxicity, in both basic neuroscience and neuropharmacological screening. Conventional methods for investigating these interactions include patch-clamp electrophysiology, which enables direct measurement of changes in ion channel activity upon dopamine receptor stimulation, providing high temporal resolution and mechanistic insights [9]. However, it is a technically demanding, low-throughput method which is unsuitable for large-scale screening applications. Fluorescence-based calcium imaging is also widely used to assess dopamine-induced intracellular signaling by measuring changes in calcium fluxes following receptor activation [10]. While this method allows visualization of cellular networks and moderately high-throughput measurements, it often requires fluorescent labeling, potentially altering cellular physiology, and it involves expensive instrumentation. Electrochemical methods such as fast-scan cyclic voltammetry (FSCV) have been employed to detect dopamine release and reuptake in neuronal preparations with sub-second resolution [11]. Although FSCV excels in sensitivity and selectivity for dopamine, it is invasive when used in vivo and does not directly measure cellular electrophysiological responses. In contrast, immobilized whole-cell-based approaches, particularly bioelectric recognition assays (BERAs), measure either the open circuit potential (OCP) or the resistance of the assayed gel-immobilized cell population using a two-electrode system [12]. BERAs offer label-free, real-time, non-destructive monitoring of dopamine–cell interactions by detecting alterations in cell electric properties upon ligand–receptor binding, and are more cost-efficient, and simpler to set up and use, compared with three-electrode amperometric or voltametric systems [13].

Arduino-based microcontroller platforms for integration into biosensor systems have gained significant attention in recent years due to their affordability, open-source architecture, and ease of programming, enabling rapid prototyping and portable analytical devices [14]. Arduino boards, such as Arduino Uno and Arduino Nano, have been widely utilized to control electrochemical sensors, optical sensors, and bioelectric impedance setups, providing real-time data acquisition and processing capabilities. For example, Arduino has been employed in the development of electrochemical biosensors for detecting environmental contaminants, glucose, and various biomolecules by controlling potentiostatic circuits and recording amperometric or voltammetric signals [15]. Regarding dopamine sensing, Mgenge et al. [16] recently reported integration of an silver chromate nanoparticle-based electroanalytical system for selective recognition of dopamine with an Arduino Uno R4 Wi-Fi module, thus facilitating transmission of real-time monitoring data to a cloud platform.

In the present study, we report the preliminary, proof-of-concept design and implementation of a two-electrode, BERA-type bioelectric biosensor based on an Arduino platform for the real-time monitoring of dopaminergic activity in gel-immobilized N2a neuroblastoma cells. We investigated the concentration-dependent effect of dopamine on the response of neuronal cells in relation to different temperatures (24 °C and 37 °C) as a key environmental factor in three-dimensional biomimetic cell cultures and also as a measure of the system’s performance under different operational parameters.

## 2. Materials and Methods

### 2.1. Chemicals, Cell Culture and Cell Immobilization

Murine neuroblastoma (N2a) cell cultures were originally provided by LGC Promochem (Middlesex, UK) and subcultured in Dulbecco’s medium with 10% fetal bovine serum (FBS), 1 μgL^−1^ antibiotics (penicillin/streptomycin) and 2 mM L-glutamine which were provided by Invitrogen (Carlsbad, CA, USA). All other reagents were purchased from Sigma-Aldrich (Taufkirchen, Germany). Following subculture N2a cells were immobilized in a 1.2% low gelling temperature (low melting temperature—LM) agarose matrix. Cell suspensions were diluted with PBS (1:10) before being plated under sterile conditions in ELISA wells and mixed with liquid agar mixture to a total volume of 200 μL per well (containing approx. 50,000 cells). Subsequently, immobilized cell cultures were either left to solidify at 24 °C or maintained in semi-liquid form at 37 °C in a CO_2_ incubation chamber.

### 2.2. Dopamine Treatment

Dopamine (DA) assays were conducted on immobilized cells on the day following gel immobilization. DA solutions in double-distilled water were prepared freshly on the day of each assay. DA was prepared at concentrations of 100 μM, 1 mM, and 10 mM using serial dilutions. Treatment of immobilized cells with DA was achieved by adding 5 μL of solution (containing DA at different concentrations) directly onto cell-containing wells under light-protected conditions. Temperature control was achieved by using a bespoke water bath.

When assaying the response of a cell population immobilized in gel, measurements reflect the cumulative contribution of cell electric properties, medium composition, and gel effects on ion traffic, as previously reported by Kintzios et al. [17]. In order to cancel out any effects that could not be attributed to cells, we used, as additional controls, microwells filled with cell-free gel and culture medium, the response of which was subtracted from the respective response of the cell-containing microwells for each measurement (see also Section 2.4. below).

### 2.3. Bioelectric Biosensor Setup

**Figure 2:**
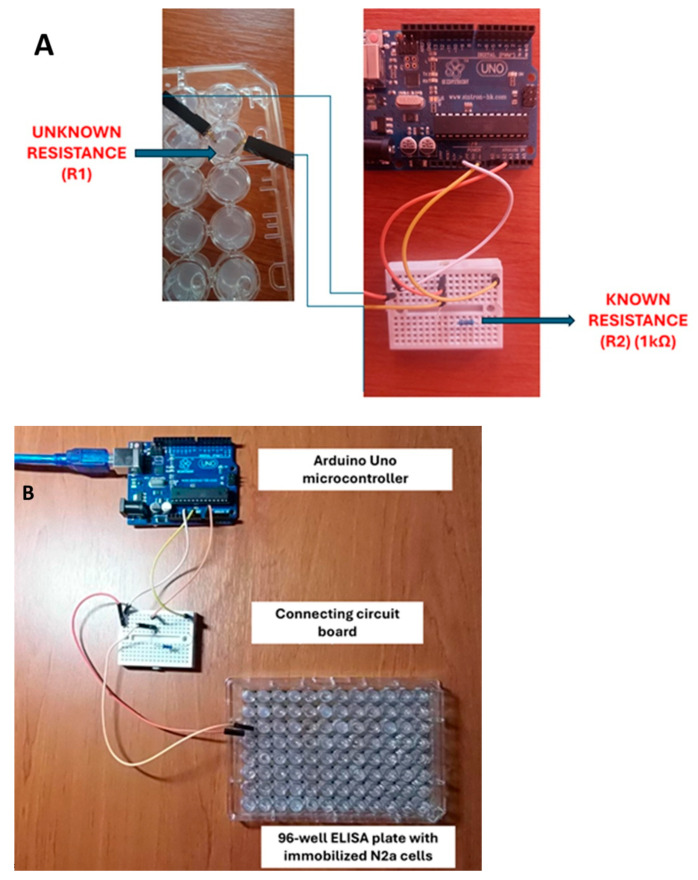
(**A**) Actual set-up of the variable resistance meter circuit based on an Arduino Uno microcontroller. The unknown, variable resistance (R1) is represented by the gel-immobilized cell system in the microwell (left) where measuring electrodes (made of Ag/AgCl) were inserted into the sides exactly opposite each well to measure the ohmic resistance. A 1 kΩ (kOhm) resistor was used as the constant, known resistance (R2). (**B**) Presentation of the complete biosensor based on an Arduino Uno microcontroller, the circuit board, and the ELISA plate containing immobilized neuroblastoma cells.

A custom Arduino-based circuit was developed to measure variable resistance (in this case, the change in resistance of the immobilized N2a cell complex after adding DA at different concentrations and at different temperatures) based on comparison with resistance of known value. According to the above provision, the known-resistance terminals (as a reference base) were connected to the analog input A0 and the ground (GDN), while the electrodes connected to the immobilized cell system (variable, unknown resistance R1) had one end connected to the 5 V power output and the other to an input of known common impedance (analog input A0). Electrodes (Ag/AgCl) were inserted into sides exactly opposite in each well to measure the bioelectric response (ohmic resistance), according to the principles of the bioelectric recognition assay (BERA). In parallel, the resistance of cell-free gel was measured in all treatment combinations. Prior to each measurement, the electrodes were rinsed with PBS and cleaned thoroughly with sterile paper. A 1 kΩ (kOhm) resistor was used as the constant resistance (R2) (Figure 2A). The system was connected to a computer on which the variable resistance measurement program was executed (see below). The system was calibrated using different precision resistors of known resistance (100 Ω, 1 kΩ and 10 kΩ) in place of the unknown resistor (R1). Sensitivity analysis was conducted by using a variable power supply (4.5–5.5 V) (Figure 2B).

The key performance parameters determined for the Arduino Uno-based resistance measurement circuit are summarized in the following Table 1:

#### Variable Resistance Measurement Program

**Figure 3:**
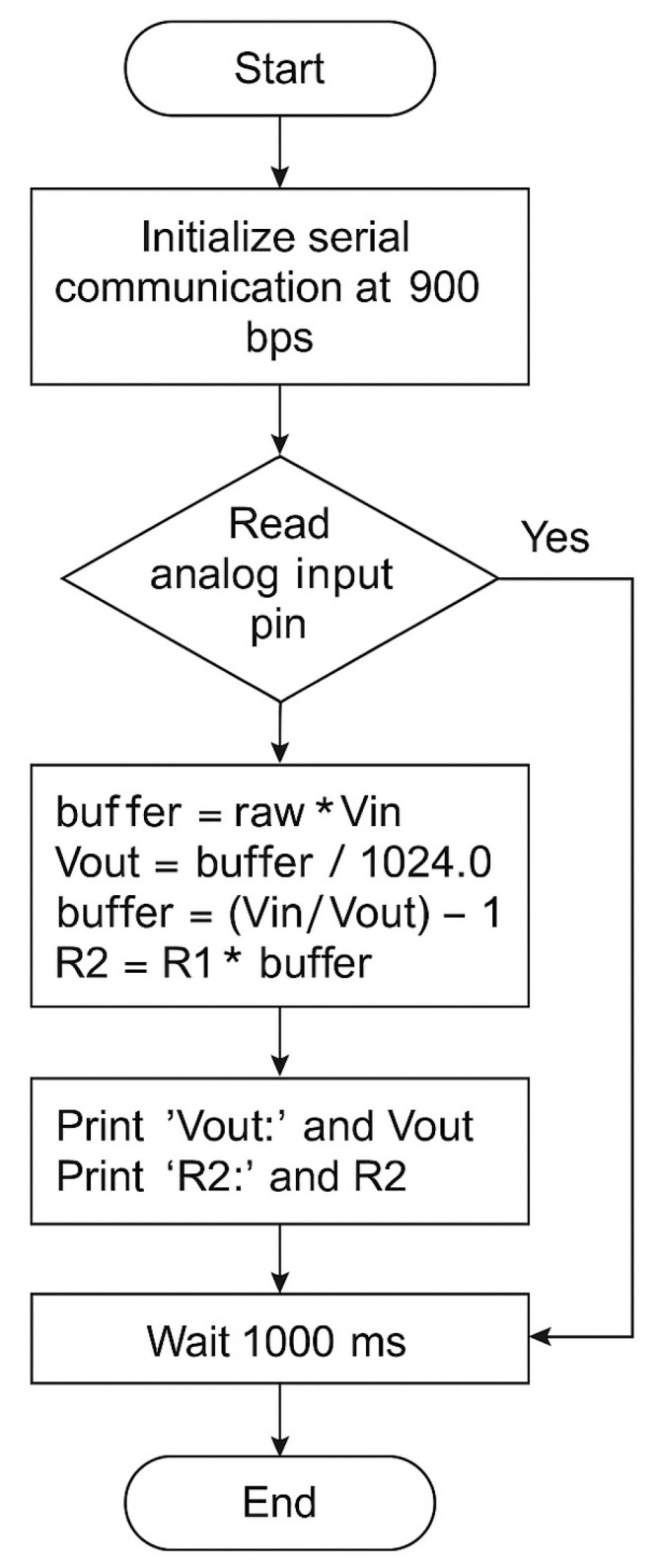
Logical flowchart of the Variable Resistance Measurement Program code used to perform variable resistance measurements through Arduino.

The program (sketch software v.1.0., Appendix A) used to perform variable resistance measurements through Arduino was developed according to the following logical flowchart (Figure 3):

Cell responses were measured directly after addition of dopamine as one-minute-long resistance time series at a frequency of 1 Hz (sampling intervals). The final readings (in ohms) were displayed in a txt file and exported into Excel for statistical analysis.

### 2.4. Statistical Analysis

For each temperature tested (24/37 °C), two individual 96-well plates containing gel-immobilized cells were used. In each plate, a set of randomly distributed wells (*n* = 24) was used for each dopamine concentration tested. In addition, experiments were conducted on eight different dates. Consequently, a total of *n* = 384 (=24 × 2 × 8) individual measurements (resistance time series) were used for each dopamine x temperature combination. As mentioned in Section 2.2. above, the resistance of cell-free agarose gel in microwells was also measured in parallel during each individual assay, and the measured value was subtracted from the cell-containing gel measurements. In this way, measurements were normalized for gel-associated resistance effects. Data was further analyzed using ANOVA to evaluate the effects of dopamine concentration and temperature on cellular response. The experimental design was set up for a randomized complete block design.

**Figure 4:**
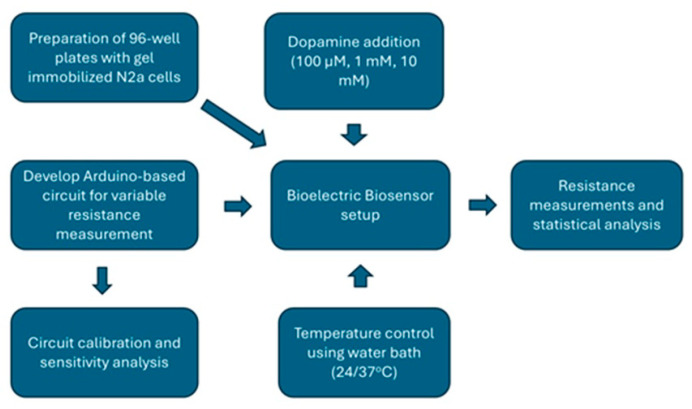
Workflow summarizing the experimental design of the study.

The experimental design is summarized in the following Figure 4:

## 3. Results and Discussion

The purpose of the present study was to conduct a preliminary investigation into the ability of a bespoke Arduino platform to monitor neural cell responses using dopamine as a model neurotransmitter. Cells were gel-immobilized in order both to provide them with a physicochemical medium which resembled their natural in vivo environment to a degree while at the same time maintaining their viability during the experimental period, and to facilitate measurements in a user-friendly, scalable way. In this respect, our research plan did not include an exhaustive range of dopamine concentrations or a detailed study of dopamine–cell interactions (e.g., by using dopamine antagonists), and only the proof-of-concept applicability of the Arduino platform in this field was assessed. That said, our work could be considered as a continuation of our previous research [13] on the bioelectric response of N2a cells to the 0–1000 μΜ range of dopamine (with an established limit of detection at 1 nM).

On the other hand, our application of room temperature (24 °C) as the alternative environmental condition deviated from standard culture conditions which allow for normal physiological cell responses. The reason for choosing this treatment was that this approach could possibly make handling of immobilized cells easier and more cost-efficient than the use of dedicated equipment (CO_2_ chamber) because it had been already demonstrated that cells could be maintained in gel for at least three weeks and continue to exhibit normal physiological functions [18], a finding which was also confirmed in the present study with an MTT viability assay.

**Figure 5:**
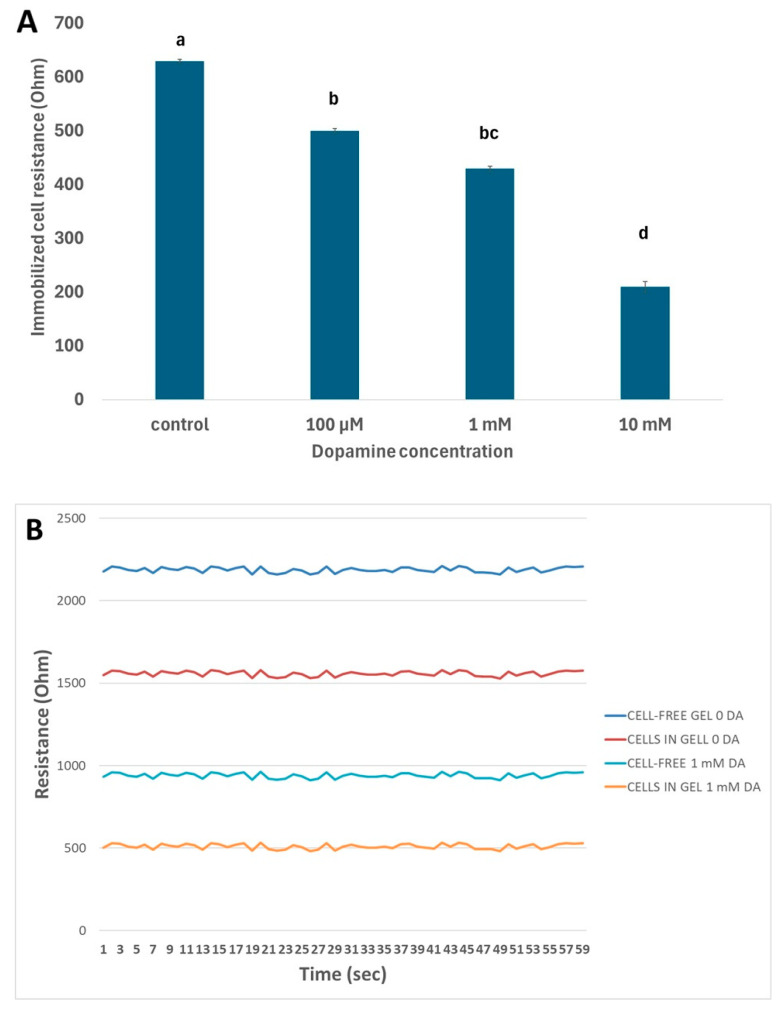
(**A**) Changes in immobilized N2a cell resistance in response to different dopamine concentrations at 24 °C. Columns indicated by different letters represent statistically different results at *p* < 0.0001 (*n* = 384 for each dopamine concentration) (values are normalized after subtracting cell-free gel responses from respective immobilized-cell responses at each dopamine concentration) (**B**) Actual traces (resistance time series) of cell-free and cell-containing gels against zero (control) and 1 mM dopamine concentrations.

**Figure 6:**
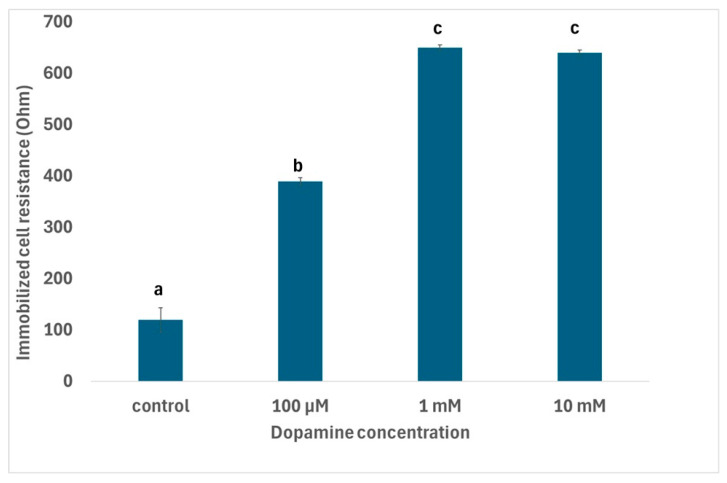
Changes in immobilized N2a cell resistance in response to different dopamine concentrations at 37 °C. Columns indicated by different letters represent statistically different results at *p* < 0.01 (*n* = 384 for each dopamine concentration).

The results showed that cells responded in specific ways at the two different temperatures: at the lower temperature (24 °C), the resistance of immobilized neuroblastoma cells declined with increasing dopamine concentrations, while most differences between concentrations were statistically significant (Figure 5A). A stable response was observed during the assay time (60 s) for each treatment, but the presence of immobilized cells affected gel resistance considerably (Figure 5B). Contrarily, an opposite pattern was observed at the higher temperature (37 °C), where the resistance of immobilized neuroblastoma cells increased with increasing dopamine concentrations (Figure 6). The observed results were reproducible, with average standard errors (over all dopamine concentrations) of 1.08 and 2.57 at 24 °C and 37 °C, respectively. These findings indicate that temperature does appear to play a particularly significant role in relation to its effect, proportionally with the concentration of dopamine, in cultured neuroblastoma cells.

In a previous study, in which a BERA-based biosensor was used to investigate the effect of dopamine on non-immobilized N2a cells, Apostolou et al. [13] found that increasing dopamine concentrations in the range from 1 μM to 1 mM was correlated with proportional reductions in cell membrane potential towards negative values and, therefore, increases in cell electric resistance, an effect which was attributed to concentration-dependent membrane hyperpolarization as a result of dopamine D2-receptor activation. Contrarily, at DA concentrations lower than 1 μΜ, an opposite pattern of membrane depolarization (increase in positive potential and decrease in cell resistance) was observed as a result of D1-receptor activation. These findings were in line with the established differential responses of D1 and D2 receptors to different ranges of dopamine concentration in conjunction with cAMP accumulation [19,20].

In the present study, N2a cells were immobilized in 1.2% LM agarose gel. This 3D cell immobilization offers a method for simulating the actual cell environment in vivo in a more realistic way than 2D cultures [21]. As already mentioned, it has been previously demonstrated that N2a cells can remain viable and proliferate in gel for at least three weeks, and continue to exhibit normal physiological functions [18]. However, the gel itself represents a diffusion barrier even for small molecules such as dopamine (molecular weight = 153,18 g/mL), meaning that cells immobilized in it will be exposed to lower DA concentrations then in a suspension culture.

In the case of the low melting temperature agarose gel used in the present study, the association between diffusion and temperature can be described by the following equation:

$$D_{37,free\;\;\approx \;}\;(D_{25,\;free})(\frac{T_{25}}{T_{37}})(\frac{n_{37}}{n_{25}})$$

where

$D_{24,\;free}$ and $D_{37,free}$ represent dopamine diffusion in water at 24 °C (~6 × 10^−10^ m^2^s^−1^) and 37 °C (~7.2 × 10^−10^ m^2^s^−1^), respectively;

*T* is the temperature (in Kelvin); and

*n* is the viscosity (m^2^s^−1^), which increases by approximately 5–7% between 24 °C and 37 °C for 1.5–2% LM agarose [22].

Provided that LM agar melts at or below 30 °C, incubating immobilized cells at 37 °C means that LM agarose no longer functions as a gel matrix; therefore, cell exposure to dopamine largely resembles conditions close to suspension culture. In such a case, cell resistance may be expected to increase with dopamine concentration due to increased membrane hyperpolarization as a result of D2-receptor activation, and this assumption was indeed corroborated by the findings obtained in the present study at 37 °C (Figure 5). Contrarily, cells at 24 °C are immobilized in a rigid, solidified gel structure, allowing only part of the applied dopamine concentration to reach them. The magnitude of this reduction in the final DA concentration reaching the cells can be described by the following equations [23]:

$$\frac{D_{24,\;gel}}{D_{37,liquid}}=\frac{D_{24,\;free}\;\times \;K_{24}}{D_{37,free}}=\frac{4.2\;\times \;10^{-10}}{7.2\;\times \;10^{-10}}=0.58$$

where

*K*_24_ = the gel permeability factor (dimensionless, <1), which is ~0.7 for small molecules in agarose gels, and increases by ~5–7% between 24 °C and 37 °C;

*D*_24_,*_gel_* = the effective diffusion coefficient of dopamine at 24 °C in gel state; and

*D*_37,*liquid*_ = the diffusion coefficient of dopamine at 37 °C in liquid (melted) state

In other words, the gel state at 24 °C reduces effective dopamine diffusion by approximately 42%, compared to the liquid state at 37 °C. Consequently, immobilized neuroblastoma cells should interact with lower dopamine concentrations, leading to activation of D1 receptors and cell membrane depolarization, which should in turn be associated with decreased cell electrical resistance. Naturally, to support the above-mentioned hypothesis on the effect of gel x temperature on dopamine diffusion to immobilized cells, a far more elaborate investigation is required, e.g., by means of radioactive labelling. However, such an analysis was beyond the scope of our essential preliminary investigation which had the practical objective of determining whether neural cell responses could in principle be monitored with the use of a simple Arduino platform.

We should also not overlook the fact that 37 °C is a temperature much more optimal for normal mammalian cell function then 24 °C, meaning that cells cultured at the second, suboptimal temperature may not be fully responsive to dopamine. Naturally, this possibility merits further investigation.

Compared to our previous research in which BERA-type assays were used for assessing N2a cell response to dopamine, the experimental approach followed in the present investigation differs in the modes of both the bioelectric properties assayed (resistance vs. open circuit potential) and the cell gel immobilization (vs. cells in suspension). In both studies, the linear correlations between biosensor response and dopamine concentration were r^2^ = 0.98 [13] and 0.95/0.87 (24/37 °C) for suspension and immobilized N2a cells, respectively, while the observed results in each experiment were highly reproducible (av. standard errors 4.4% vs. 1.8% in the present study). Therefore, it is reasonable to assume that the novel experimental approach presented in this report could be used as a reliable alternative to the previously established bioelectric assays for the same monitoring purpose.

## 4. Conclusions

In the present study we demonstrate, as proof of concept, that integrated microcontroller systems such as Arduino, when combined with a simple bioelectric recording set-up, can be used as a cost-efficient and user-friendly platform for screening the bioactivity of neurotransmitters, as a first step before employment of more elaborate and sophisticated analytical tools. Integration of such a design with wireless modules (e.g., Bluetooth or Wi-Fi) facilitates remote monitoring applications, enhancing usability in point-of-care diagnostics and in-field analyses [24]. Additionally, Arduino platforms can be combined with smartphone applications to visualize and store biosensor data in real time, expanding their accessibility in resource-limited settings [25]. That said, the present work could form the basis for further developing a novel biosensor tool for dopamine detection after it is tested against a wider range of dopamine concentrations, along with various dopamine agonists and antagonists, after determining its specificity, selectivity, and sensitivity.

## Figures and Tables

**Table 1:** Key performance parameters of the Arduino Uno-based resistance measurement circuit.

Parameter	Estimated Range/Value	Comments
ADC resolution	10-bit (4.88 mV steps)	Limited, nonlinear for resistance calculation
Resistance resolution	±5–100 Ω (depends on actual R)	Best near R ≈ R_1_ (1 kΩ)
Gain	1 × (no amplification)	Can be modified with op-amp
Noise floor	±2–5 ADC counts (~±10–25 mV)	High in noisy environments
Filter options	Not implemented by default	Add software/hardware filters for better SNR

## Data Availability

The raw data (3.072 cell resistance time series) supporting the conclusions of this article will be made available by the authors on request.

## Abbreviations

The following abbreviations are used in this manuscript:

ADHD	Attention-deficit hyperactivity disorder
BERA	Bioelectric recognition assay
DA	Dopamine
FSCV	Fast-scan cyclic voltammetry
HPLC	High-performance liquid chromatography
LM	Low melting temperature

## Appendix A. Variable Resistance Measurement Program Code (Sketch Software)

int analogPin = 0;

int raw = 0;

int Vin = 5;

float Vout = 0;

float R1 = 1000;

float R2 = 0;

float buffer = 0;

void setup()

{

Serial.begin (9600);

}

void loop()

{

raw = analogRead (analogPin);

if(raw)

{

buffer = raw * Vin;

Vout = (buffer)/1024.0;

buffer = (Vin/Vout) −1;

R2 = R1 * buffer;

Serial.print (“Vout:”);

Serial.println (Vout);

Serial.print (“R2:”);

Serial.println (R2);

delay (1000);

}

}
